# Comparing Eight Prognostic Scores in Predicting Mortality of Patients with Acute-On-Chronic Liver Failure Who Were Admitted to an ICU: A Single-Center Experience

**DOI:** 10.3390/jcm9051540

**Published:** 2020-05-20

**Authors:** Bo-Huan Chen, Hsiao-Jung Tseng, Wei-Ting Chen, Pin-Cheng Chen, Yu-Pin Ho, Chien-Hao Huang, Chun-Yen Lin

**Affiliations:** 1 Division of Hepatology, Department of Gastroenterology and Hepatology, Chang-Gung Memorial Hospital, Linkou Medical Center, Taoyuan 333, Taiwan; spring03258@gmail.com (B.-H.C.); weiting1972@gmail.com (W.-T.C.); Pin_chang0730@hotmail.com (P.-C.C.); ho12345678.tw@yahoo.com.tw (Y.-P.H.); huangchianhou@yahoo.com.tw (C.-Y.L.); 2 Biostatistics Unit, Clinical Trial Center, Chang-Gung Memorial Hospital, Linkou Medical Center, Taoyuan 333, Taiwan; allebjht@gmail.com; 3 College of Medicine, Chang-Gung University, Taoyuan 333, Taiwan; 4 Graduate Institute of Clinical Medical Sciences, College of Medicine, Chang-Gung University, Taoyuan 333, Taiwan

**Keywords:** acute-on-chronic liver failure (ACLF), APACHE III, CLIF-OFs, CLIF-C ACLF, specialized liver intensive care unit, Time-dependent ROC curve

## Abstract

Limited data is available on long-term outcome predictions for patients with acute-on-chronic liver failure (ACLF) in an intensive care unit (ICU) setting. Assessing the reliability and accuracy of several mortality prediction models for these patients is helpful. Two hundred forty-nine consecutive patients with ACLF and admittance to the liver ICU in a single center in northern Taiwan between December 2012 and March 2015 were enrolled in the study and were tracked until February 2017. Ninety-one patients had chronic hepatitis B-related cirrhosis. Clinical features and laboratory data were collected at or within 24 h of the first ICU admission course. Eight commonly used clinical scores in chronic liver disease were calculated. The primary endpoint was overall survival. Acute physiology and chronic health evaluation (APACHE) III and chronic liver failure consortium (CLIF-C) ACLF scores were significantly superior to other models in predicting overall mortality as determined by time-dependent receiver operating characteristic (ROC) curve analysis (area under the ROC curve (AUROC): 0.817). Subgroup analysis of patients with chronic hepatitis B-related cirrhosis displayed similar results. CLIF-C organ function (OF), CLIF-C ACLF, and APACHE III scores were statistically superior to the mortality probability model III at zero hours (MPM0-III) and the simplified acute physiology (SAP) III scores in predicting 28-day mortality. In conclusion, for 28-day and overall mortality prediction of patients with ACLF admitted to the ICU, APACHE III, CLIF-OF, and CLIF-C ACLF scores might outperform other models. Further prospective study is warranted.

## 1. Introduction

Acute-on-chronic liver failure (ACLF) is a recently recognized syndrome characterized by acute cirrhotic decompensation, organ failure, and marked short-term mortality (28-day mortality of 30%~40%) [1] as defined in the CANONIC study [2]. This syndrome usually requires admission to an intensive care unit [3], yet in contrast to cirrhotic decompensation, which is considered irreversible given the loss of regenerative potential, ACLF is reversible if the precipitating event can be managed [4]. Thus, intensive care with expertise in both hepatology and critical care is required for a chance at recovery [3]. Patients with ACLF who do not improve with supportive measures are potential candidates for liver transplantation (LT). However, these patients are also at higher risk for rapid clinical deterioration. Hence, it is crucial that such high-risk cases are identified and prioritized for LT accordingly. Clinical presentation and prognosis for patients without organ failure are different [2]. There is, therefore, a need to identify the most reliable prognostic model as well as to establish cutoff values for these critically ill patients.

Several prognostic scores have been developed for patients critically ill or with ACLF. The sequential organ failure assessment score, also known as the sepsis organ failure assessment score (SOFAs), is widely used to track patient status during intensive care in order to determine the extent of organ dysfunction and failure over time [5]. The CANONIC study applied this score to cirrhotic patients and subsequently proposed the chronic liver failure SOFA score (CLIF SOFAs) as a diagnostic criterion for ACLF [6]. One previous study found that both CLIF-SOFAs and APACHE III recorded on the day of intensive care unit (ICU) admittance were excellent prognostic tools for severe cirrhosis [7]. Since ACLF is distinct from acute cirrhotic decompensation [2], it is valuable to compare these scores among patients with ACLF admitted to an ICU.

The CLIF consortium organ function score (CLIF-C OFs) was developed to simplify and improve the accuracy of ACLF diagnosis and grading [2]. The CLIF consortium acute-on-chronic liver failure score (CLIF-C ACLFs) was subsequently proposed based on the CLIF-C OF scores of patients hospitalized at a single ICU [8]. Another multicenter pilot survey in Japan also demonstrated the utility of the European Association for the Study of the Liver (EASL)-CLIF consortium criteria in outcome prediction [9]. In addition, several generations of the acute physiology and chronic health evaluation (APACHE) score have been developed, and together form a validated ICU prognostic system based on illness severity and a focus on the general health of adult, critically ill, patients [10,11,12,13]. Kuzniewicz et al. demonstrated that the APACHE model provided an excellent predictive accuracy in ICU risk-adjusted mortality (AUC = 0.892) [14]. Two additional ICU mortality prediction models, the mortality probability model III at zero hours (MPM0-III) and the simplified acute physiology score (SAPs) III, use alternative risk-adjustment methods to assess mortality [15,16,17]. Other commonly used prognostic systems for patients with liver cirrhosis include the Child–Turcotte–Pugh (CTP) score, and the model for end-stage liver disease (MELD) score that have been used as major considerations when allocating donor livers in the United States since February of 2002 [18].

Few studies have compared the ICU prognostic scores to liver scores for those ACLF patients. In clinical practice, which score is better at predicting overall mortality remains controversial. Therefore, the aim of this study was to compare the eight prognostic scores among patients with ACLF and a history of admittance to an ICU in a viral hepatitis endemic state like Taiwan by a single-center experience. Each score was calculated according to its own criteria, and only data obtained upon and/or within the first 24 h of the first ICU admittance were used. The ability of each measure to predict mortality was then compared with respect to several pre-defined follow-up periods. Table S1 was established to list the variables that each score takes into consideration.

## 2. Methods

### 2.1. Patient Selection

This retrospective study was approved by the Ethics Committee established by the Chang Gung Medical Foundation Institutional Review Board (IRB number: 201701810B0). Inclusion criteria consisted of cirrhotic patients meeting diagnosis standards for ACLF [2] and with a record of admittance to our hospital’s 18-bed hepatogastroenterology ICU for intensive monitoring and/or treatment between December 2012 and March 2015. Our exclusion criteria were age below 18 years, pregnancy, non-curable hepatocellular carcinoma, an ACLF score of 0 (absence of ACLF), and patients (nine in total) who received orthotopic liver transplantation (OLT) during follow-up. For patients with repeat admittance, only the initial ICU stay was analyzed to avoid repeat weighing.

### 2.2. Follow-Up Periods

Patients were enrolled between December 2012 and March 2015 and were tracked until February 2017.

### 2.3. ACFL Diagnosis and Grading

Patients in our study fulfilled the European Association for the Study of the Liver (EASL)-CLIF consortium diagnostic criteria for ACLF diagnosis. EASL-CLIF consortium criteria were also used for assessing ACLF severity [2,8]. In brief, ACLF 1: Single kidney failure or single liver/coagulation/circulatory/lung failure associated with a serum creatinine level of 1.5–1.9 mg/dL and/or hepatic encephalopathy of West-Heaven grade 1 or 2; ACLF 2: Failure of two organs; ACLF 3: Failure of three or more organs. The diagnosis of liver cirrhosis was based on either histopathological diagnosis or a composite of compatible clinical features, laboratory tests, endoscopy, and radiologic imaging.

### 2.4. Data Collection

Data on patient demographics, oxygenation support, ventilator record, hemodynamic status (heart rate, blood pressure, body temperature), Glasgow coma scale (GCS) score, peripheral complete blood count and sedimentation rate, blood chemistry tests, urine output, recent immunosuppression events, etiology of cirrhosis, and survival time were retrospectively obtained from medical charts. All data were collected upon and/or within 24 h of admission to the ICU.

### 2.5. The Primary Endpoint and the Pre-Defined Follow-Up Periods

The primary study endpoint was overall survival. It was defined as censored at 3.5 years if patients were followed-up with for over 42 months. Mortality predictions at time points of 28, 90, 180, and 365 days of follow-up were also calculated.

Patient follow-up condition after hospital discharge was determined by analysis of chart records and/or by telephone interview.

### 2.6. Patient Treatment

Treatment of ACLF patients with hepatitis B virus (HBV)-related cirrhosis and a history of taking antiviral agents met the latest Asia-Pacific Association for the Study of the Liver (APASL) guidelines [19,20,21]. Treatment of ACLF conformed to APASL and EASL guidelines [22,23].

### 2.7. Statistical Analysis

Continuous variables are expressed as mean ± standard deviation (SD) or median (interquartile range (IQR)) in accordance with their distribution: normal or skewed. A two-sample independent t-test or Mann–Whitney U test was used to compare continuous variables between survivors and non-survivors, respectively. Categorical variables are described as frequencies and percentages, with the Chi-square test for comparison. When it came to a situation where more than 20% of data cells presented an expected frequency of <5, Fisher’s exact test was substituted for the Chi-square test.

Time-dependent receiver operating characteristic (ROC) analysis was used to compare the prediction performance among these clinical indexes. The areas under the ROC curves (AUROC) indicate the predictive accuracy in overall survival. The Hanley and McNeil test [24] was used to conduct inter-measure comparisons. The optimal cutoff values were determined by the point on which the clinical index at one year exceeded 70% in sensitivity with the highest specificity. Whereas the cutoff values for futility were determined by the critical value on the 28th day, on which up to 80% mortality occurred.

All statistical analyses were done by STATA15.0 and figures were illustrated by R3.5.0. A *p* value of <0.05 was considered statistically significant.

## 3. Results

### 3.1. Demographics

Two hundred forty-nine consecutive patients with ACLF and past admittance to the liver ICU between December 2012 and March 2015 were enrolled and were tracked until February 2017. Table 1 shows the basic characteristics of these 249 patients, which were predominantly middle-aged (55 ± 13 years) and male (74%). The etiologies of cirrhosis were mostly from alcohol abuse (98 patients, 39%) or related to hepatitis B virus infection (91 patients, 37%). Gastrointestinal bleeding (127 patients; 57%) accounted for most of the indications for ICU admission, and the average ICU stay lasted 27 days. The value of each scoring system between survivor and non-survivor groups during the overall follow-up period and other clinical parameters are described. In terms of ACLF patient assessment, Grade 3 ACLF made up the largest group (43%), followed by Grade 1 (38%) and Grade 2 (19%). In-hospital mortality was 43%, and mortality rates at 1, 3, 6, and 12 months were 25%, 43%, 52%, and 58%, respectively. The overall mortality rate (mean follow-up duration of 416 days (1–1792)) was 72%.

### 3.2. Comparisons of the Outcome Prediction Strengths of the Eight Models by AUROC Analysis

**Figure 1:**
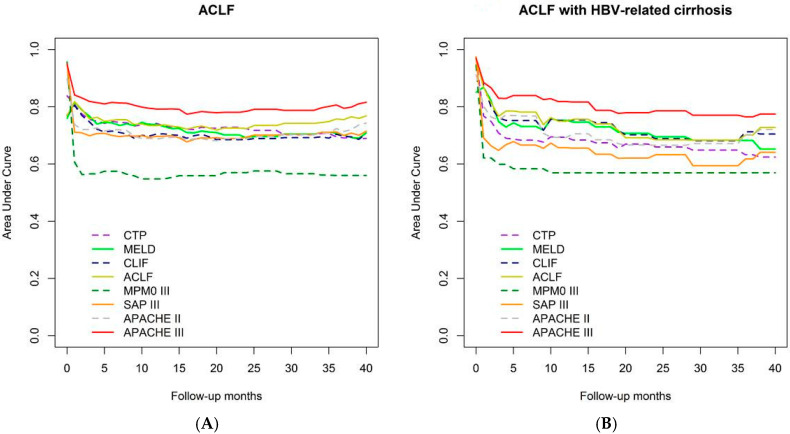
Comparison of the 8 prognostic scores to predict overall mortality by time-dependent area under the receiver operating characteristic curve (AUROC). (**A**) Total ACLF patients (redline: APACHE III); (**B**) ACLF patients with HBV-related cirrhosis (redline: APACHE III).

**Figure 2:**
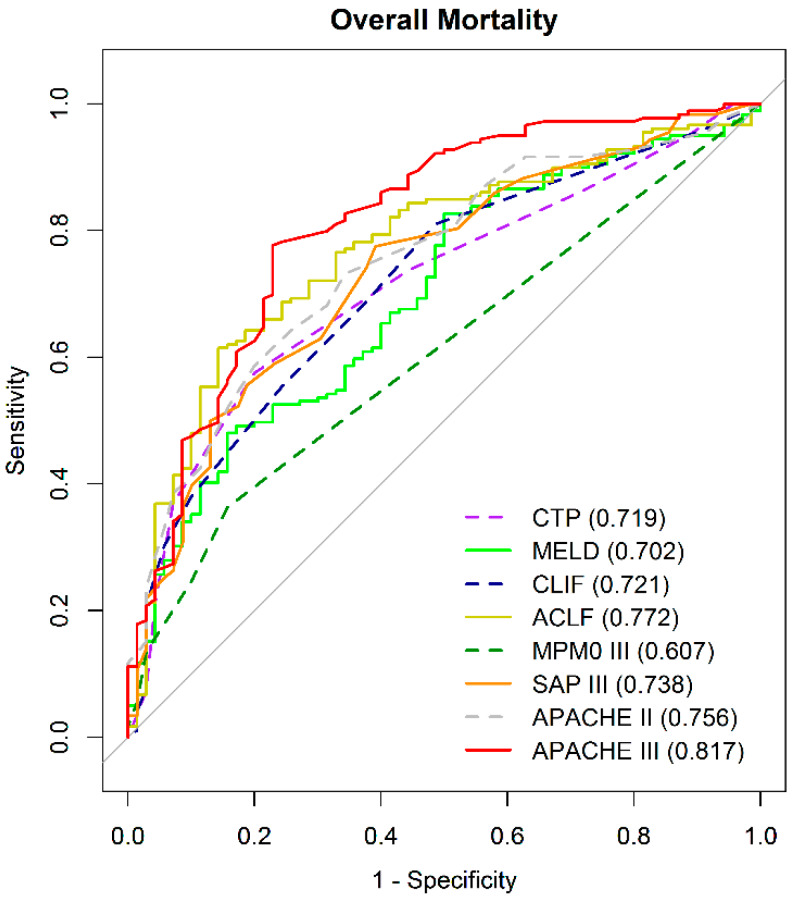
Comparison of the 8 prognostic scores to predict overall mortality by AUROC at specific time point.

**Figure 3:**
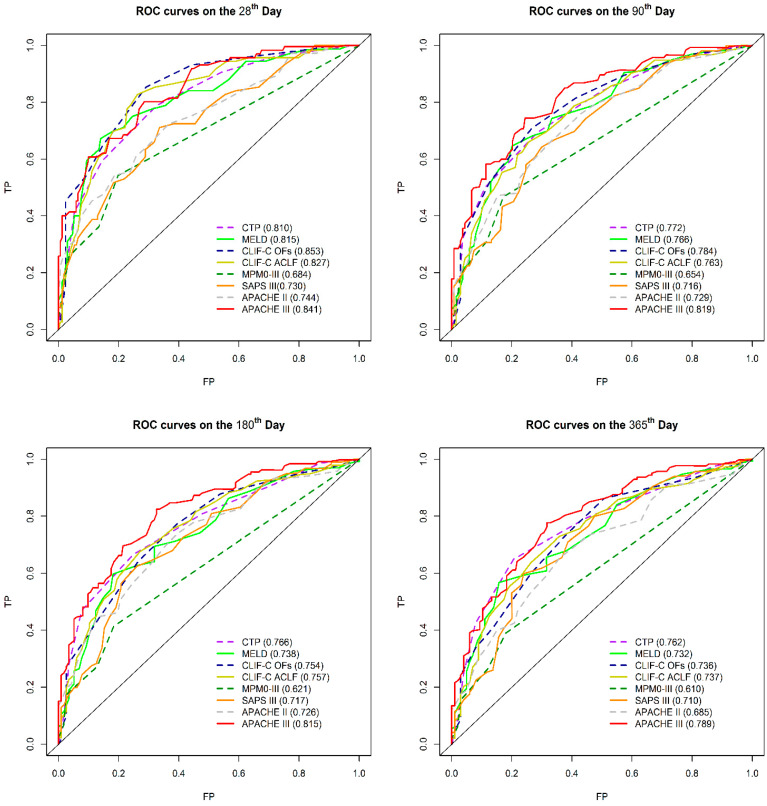
Comparison of prognostic scores to predict 28-day, 90-day, 180-day, and 365-day mortality by AUROC.

We compared the AUROCs of the eight models concurrently to predict overall mortality, which was defined as censored at 3.5 years if patients were followed-up with for over 42 months (mean duration 416 days; minimum–maximum: 1–1792 days) as shown in Figure 1 and Figure 2. In addition, comparisons of the eight models in predicting mortality on the 28th, 90th, 180th, and 365th days are shown in Figure 3. The detailed description and explanation are presented in the following paragraphs.

#### 3.2.1. Time-Dependent AUROC Comparison of Overall Mortality Prediction Strength

By time-dependent ROC analysis of the eight models predicting overall mortality among 249 ACLF patients, the AUROCs of APACHEIII and CLIF-C ACLF scores were consistently higher than those of the other six models after 28 days as well as for the overall follow-up period (Figure 1A).

Data from 91 patients with chronic hepatitis B-related cirrhosis underwent subgroup analysis given the high HBV prevalence in Taiwan [25], and patient demographics are shown in Table S2. Time-dependent ROC analysis of the models reveals that the AUROCs of APACHE III and CLIF-C ACLF scores were consistently higher than that of the other six models after 28 days as well as for the overall follow-up period in patients with chronic hepatitis B-related cirrhosis (Figure 1B).

#### 3.2.2. Comparison of the Overall Mortality Prediction Strength by AUROC at Specific Time Point

The AUROCs (95% confidence intervals (CIs)) of CTP, MELD, CLIF-C OFs, CLIF-C ACLF, MPM0-III, SAP III, APACHE II, and APACHE III for overall mortality prediction were 0.719 (0.652–0.785), 0.702 (0.631–0.772), 0.721 (0.653–0.790), 0.772 (0.708–0.836), 0.607(0.552–0.663), 0.739 (0.671–0.806), 0.756 (0.692–0.820), and 0.817 (0.756–0.878), respectively (Table 2). APACHE III and CLIF-C ACLF scored had the highest AUROC, significantly superior to that of CTP, MELD, CLIF-OF, MPM0-III, and SAP III (Table 2 and Figure 2).

#### 3.2.3. Comparison of 28-Day, 90-Day, 180-Day, and 365-Day Mortality Predictions by AUROC

In predicting the 28-day mortality, there was significant difference between APACHE III, CLIF-C OF, CLIF-C ACLF, and MELD in pairwise comparison. However, these scores were all statistically superior to MPM0-III and SAPs III (Figure 3 and Table 3).

Furthermore, APACHE III displayed the highest AUROC and was significantly superior to that of MPM0-III, SAP III, and APACHE II in predicting the 90-day, 180-day, and 365-day mortality. There were no significant differences between APACHE III, CLIF-C OF, CLIF-C ACLF, CTP, and MELD in mortality prediction at these time points. However, they were all statistically superior to MPM0-III and SAPs III (Figure 3).

#### 3.2.4. The Optimal and Futility Cutoff Values for APACHE III and CLIF-C ACLF Scores

According to our cutoff determination, mentioned in the Statistical Method Section, we found the optimal cutoffs were 79 for the APACHE III score and 47 for the CLIF-C ACLF score (Table 4). The optimal cutoffs assure over 70% sensitivity, and the specificity increases with time. The cutoffs for futility were also sought for APACH III and CLIF-C ACLF. Assuring that 80% mortality would happen if the value exceeded the cutoff point, the futility cutoffs were determined as 125 for the APACHE III score and 71 for the CLIF-C ACLF score. Our CLIF-C ACLF score’ futility cutoff approximates what Cornelius Engelmann et al. found that CLIF-C ACLF scores ≥70 are associated with futility [26].

## 4. Discussion

In this study, we compared eight different prognostic scoring systems simultaneously for patients with ACLF who were admitted to our hepatogastroenterology ICU, which provides a single-center experience in a chronic viral hepatitis endemic state. The results showed that APACHE III and CLIF-C ACLF scores were significantly superior to other models in predicting overall mortality as determined by time-dependent ROC curve analysis (AUROC: 0.817). Subgroup analysis of patients with chronic hepatitis B-related cirrhosis displayed similar results. CLIF-C OFs, CLIF-C ACLF, and APACHE III were statistically superior to MPM0-III and SAPs III in predicting 28-day mortality. In summary, for 28-day and overall mortality prediction of patients with ACLF admitted to the ICU, APACHE III, CLIF-OF, and CLIF-C ACLF scores might outperform other models. However, if constrained by cost and manual data collection, CLIF-C ACLF offers a good and simple alternative. Assuring that 80% mortality would happen if the value exceeded the cutoff point, the futility cutoffs were determined as 125 for the APACHE III score and 71 for the CLIF-C ACLF score. Our CLIF-C ACLF score’s futility cutoff approximates what Cornelius Engelmann et al. found, with CLIF-C ACLF scores ≥70 being associated with futility [26]. These cutoffs could aid clinicians in deciding, for example, when to stop therapies or plan different strategies such as fast OLT assessment. Further prospective study is warranted to assess the value of these scores for better liver transplantation priority assessment.

Finding the most accurate prognostic tool for patients with ACLF, especially for those admitted to the ICU, is crucial. This patient population exhibits high short-term and long-term mortality [2,4,8,27], so more aggressive treatment modalities such as liver transplantation are often considered. However, due to the shortage of donor organs in Taiwan [28], prioritizing liver transplantation for those with ACLF without contraindications could improve short-term and long-term survival. A previous study showed that both CLIF-SOFA and APACHE III scores recorded on the first day of ICU admission were excellent prognostic tools for critically ill cirrhotic patients admitted to the ICU [7]. These scores were only used to predict 6-month mortality, and ACLF is a distinct condition from acute decompensation due to cirrhosis [2]. Hence, we found it intriguing to compare the eight commonly used prognostic scores simultaneously in patients with ACLF admitted to the ICU.

ACLF has emerged as a unique syndrome characterized by acute deterioration of health with compensated or relatively stable decompensated cirrhosis, the frequent need for organ support, and an association with high short-term mortality. The CLIF-SOFAs and CLIF-C OFs are new scoring systems for this patient population [1,2,8], but few studies have assessed these scores in predicting long-term outcomes. Furthermore, various descriptive and prognostic evaluation/scoring systems have been developed over the past decade [10,12,29,30,31] and are widely used in the ICU setting to assess resource use, predict outcomes, characterize disease severity, and evaluate degree of organ dysfunction [12]. However, the accuracy of these systems among different patient subgroups has recently been called into question [29].

In this single-center retrospective study, patients displayed a high overall mortality rate (72% during the mean follow-up period of 416 days). Among the available eight commonly used prognostic scoring systems, APACHE III score and CLIF-C ACLF score were significantly superior to the other five models in predicting overall mortality as determined by time-dependent ROC curve analysis. In predicting the 28-day mortality, APACHE III showed no significant difference to CLIF-C OF CLIF-C ACLF, and MELD. However, they were all statistically superior to MPM0-III and SAPs III. Furthermore, APACHE III displayed the highest AUROC and was significantly superior to MPM0-III, SAPs III, and APACHE II in predicting the 90-day, 180-day, and 365-day mortality. In fact, APACHE was developed based on ICU data in the U.S. and was upgraded to its fourth version [31] in 2006. APACHE IV predictions of hospital mortality have good discrimination and calibration, but are impractical in the absence of accompanying spreadsheets [31]. Therefore, APACHE II and its extension APACHE III continue to be used in clinical practice [32]. Another study demonstrated APACHE III and IV as possessing similar discriminatory capability [33]. Given these reasons, APACHE III is the outcome prediction tool of choice at our hepatogastroenterology ICU given that data for APACHE IV remains unavailable.

We also compared APACHE III with other adult intensive care unit prognostic systems such as MPM0-III and SAP III. Our results demonstrated that APACHE III predicts outcomes better than SAPS III, which in turn predicts outcomes better than MPM 0-III. This is in line with previous retrospective studies [7,33,34].

Our finding that CLIF-C OF, CLIF-C ACLF, and APACHE III scores were statistically superior to MPM0-III and SAP III scores in predicting 28-day mortality corresponds to the following studies. One study reported that the CLIF-SOFAs could more accurately predict short-term mortality in patients with acute decompensated alcoholic cirrhosis than five existing scoring systems for end-stage liver disease such as the MELD-based systems and CTP [35]. Another study revealed that the CLIF-C ACLFs may be more useful for predicting 28-day and 90-day mortality in alcohol-related ACLF than CTP, MELD, and MELD-sodium scores [36]. Furthermore, one study on patients with HBV-related ACLF showed that the CLIF-OFs is superior to MELD, CLIF-SOFA, and CLIF-C ACLF scores in predicting 28-day mortality [37].

Previous studies indicated that ACLFs is a clinically and pathophysiologically distinct disease in patients with HBV [38]. Subgroup analysis by time-dependent ROC of the eight models in predicting overall mortalities for patients with ACLF and chronic hepatitis B-related cirrhosis demonstrated that, except for CLIF-C ACLF, the AUROC of APACHE III was consistently higher than that of the other seven models after 28 days and until the end of the follow-up period. In fact, improvement to the accuracy of prognostication by HBV-ACLF is critical to the early identification of candidates for LT [39]. Hence, APACHE III could aid in identifying HBV-ACLF patients that require urgent and aggressive management or provide a basis for discussing withdrawal of care with family members in chronic viral hepatitis endemic regions such as Taiwan. It is worth noting that the prevalence of HBV-related cirrhosis in our ACLF patient cohort was 37%, which is higher than in the CANONIC study, but lower than those with alcohol-related cirrhosis (39%). This may be due to the good control of chronic hepatitis B infection in Taiwan in recent years [40].

There were several limitations to the study. First, data acquisition at different time-points during the hospital course might impact the predictive abilities of the eight prognostic scoring systems, for example, the CLIF-C ACLF score [8]. Some scholars advocate data from days 3–7 following diagnosis [41], while others use data acquired on day 1 of ICU admittance for assessing critically ill patients. We used data acquired within day 1 of ICU admittance for analysis because it was more unanimous and convenient. Second, some of the prognostic systems, such as CTP and MELD, were not created for patients with ACLF but were still listed herein because they are commonly used clinically for evaluating the prognosis of patients with end-stage liver disease. Our results indicate that other prognosis systems, such as APACHE-III, should be preferentially used for patients with ACLF admitted to the ICU. Third, APACHE IV was not compared in the study given that without its prepared spreadsheets, it is impractical to use [31].

## 5. Conclusions

Among the eight commonly used prognostic scoring systems for patients with ACLF admitted to our ICU, the APACHE III score and CLIF-C ACLF score were significantly superior to other models in predicting overall mortality as determined by time-dependent ROC curve analysis according to our single-center experience in a chronic viral hepatitis endemic state. In predicting the 28-day mortality, APACHE III showed no significant difference to CLIF-C OF, CLIF-C ACLF, and MELD. However, they were all statistically superior to MPM0-III and SAPs III. Subgroup analysis of 91 ACLF patients with HBV-related cirrhosis displayed similar results. Therefore, from a research perspective, it is an interesting finding that APACHE III performed as well as CLIF-C ACLF based on ICU admission day data in predicting overall and other time-point mortality for those patients with ACLF admitted to the ICU. However, if constrained by cost and manual data collection, CLIF-C ACLF offers a good and simple alternative. Further prospective study is warranted to assess the value of these scores for better liver transplantation priority assessment.

## Figures and Tables

**Table 1:** Demographics of 249 patients with acute-on-chronic liver failure (ACLF) admitted to the intensive care unit (ICU).

Patients’ Characteristics	All Patients	Survivors	Non-Survivors	*p* Value
	(249 Patients)	(70 Patients)	(179 Patients)	
Age (mean ± SD)	55 ± 13	50 ± 10	57 ± 14	<0.001
Gender = male	184 (74%)	51 (73%)	133 (74%)	0.81
Etiology				
HBV	91 (37%)	22 (31%)	69 (38%)	0.294
HCV	34 (14%)	5 (7%)	29 (16%)	0.061
ALC	98 (39%)	33 (47%)	65 (36%)	0.116
HCV + ALC	10 (4%)	5 (7%)	5 (3%)	0.117
Other ^†^	16 (6%)	5 (7%)	11 (6%)	0.773
Clinical parameters:				
Arterial PH	7.4 (7.3–7.5)	7.4 (7.4–7.5)	7.4 (7.3–7.5)	0.110
PaO2/FiO2 200–300	194	61 (87%)	132 (73%)	0.023
PaO2/FiO2 < 200	55	9 (13%)	47 (27%)	0.023
MAP (mmHg)	88 (76–102)	87 (80–104)	88 (76–101)	0.581
Temperature (℃)	36.6 (36.0–37.3)	36.9 (36.3–37.6)	36.5 (35.8–37.2)	0.001
Respiratory rate (/min)	19 (16–22)	18 (16–20)	19 (17–23)	0.082
Use of vasopressors	140	35 (50%)	105 (59%)	0.216
HE I-II	92	17 (24%)	75 (42%)	0.01
HE III-IV	36	7 (10%)	29 (16%)	0.211
White cell count (×1000/μL)	9.3 (6.3–12.9)	8.3 (5.8–10.9)	9.6 (6.8–15.1)	0.010
Hematocrit (mg/dL)	26.7 (22.7–31.2)	27.5 (22.3–31.5)	26.7 (22.7–31.2)	0.907
INR	1.7 (1.4–2.2)	1.5 (1.3–1.8)	1.8 (1.5–2.4)	<0.001
Serum bilirubin (mg/dL)	3.6 (1.7–9.5)	2.2 (1.4–4.1)	4.2 (2.0–12.8)	<0.001
Serum creatinine (mg/dL)	1.22 (0.82–2.07)	0.84 (0.66–1.38)	1.35 (0.95–2.24)	<0.001
Serum sodium (mEq/L)	138 (134–142)	138 (136–141)	138 (133–142)	0.459
Serum glucose (mg/dL)	172 (135–229)	154 (122–198)	179 (139–241)	0.013
Albumin (g/dL)	2.65 (2.29–2.98)	2.87 (2.54–3.20)	2.60 (2.20–2.88)	0.047
Mechanical ventilation use	85	17 (24%)	68 (38%)	<0.001
ACLF grades of EASL-CLIF consortium:				
ACLF 1	106 (43%)	43 (61%)	63 (35%)	0.35
ACLF 2	48 (19%)	11 (16%)	37 (21%)	0.066
ACLF 3	95 (38%)	16 (23%)	79 (44%)	<0.001
Indications for ICU admission:				
Gastrointestinal bleeding	127 (57%)	43 (61%)	84 (47%)	0.040
Hepatic encephalopathy	35 (12%)	14 (21%)	21 (12%)	0.092
Sepsis	56 (22%)	9 (13%)	47 (26%)	0.023
Other	31 (13%)	4 (5%)	27 (15%)	0.053
Score on admission to ICU median (IQR):				
CTP	9.0 (8.0–11.0)	8.0 (7.0–9.0)	10.0 (8.0–11.0)	<0.001
MELD	23.0 (18.0–30.0)	18.5 (16.0–25.0)	25.0 (19.0–34.0)	<0.001
CLIF-C OF	10.0 (8.0–12.0)	8.0 (8.0–10.0)	11.0 (9.0–13.0)	<0.001
CLIF-C ACLF	49.2 (41.8–60.5)	41.8 (37.5–47.9)	52.6 (46.3–63.4)	<0.001
SAP III	51.0 (46.0–59.0)	47.0 (43.0–51.0)	54.0 (48.0–63.0)	<0.001
MPM0-III	0.0 (0.0–1.0)	0.0 (0.0–0.0)	0.0 (0.0–1.0)	0.001
APACHE II	16.0 (12.0–22.0)	13.0 (9.0–16.0)	18.0 (13.0–23.0)	<0.001
APACHE III	81.0 (61.0–103.0)	55.0 (41.0–70.0)	87.0 (73.0–108.0)	<0.001

IQR: Interquartile range (Q1–Q3); ^†^ other etiologies of cirrhosis include autoimmune hepatitis, fatty liver related, etc. HE: hepatic encephalopathy; HBV: chronic hepatitis B infection-related cirrhosis; HCV: chronic hepatitis C infection-related cirrhosis; ALC: alcohol-related cirrhosis.

**Table 2:** Comparison of the 8 prognostic scores to predict overall mortality by AUROC.

		AUROC (95%CI)		Pairwise Sig. Mark							
				A	B	C	D	E	F	G	H
CTP	A	0.719	(0.652–0.785)								
MELD	B	0.702	(0.631–0.772)								
CLIF-C OF	C	0.721	(0.653–0.790)								
CLIF-C ACLF	D	0.772	(0.708–0.836)								
MPM0-III	E	0.607	(0.552–0.663)								
SAP III	F	0.739	(0.671–0.806)								
APACHE II	G	0.756	(0.692–0.820)								
APACHE III	H	0.817	(0.756–0.878)								

AUROC: area under receiver operating characteristic curve; The yellow color regions reflect significant difference in pairwise comparison at significance level α = 0.05 and is more important than the black ones.

**Table 3:** Comparison of the 8 prognostic scores to predict 28-day mortality by AUROC.

		AUROC (95%CI)		Pairwise Sig. Mark							
				A	B	C	D	E	F	G	H
CTP score	A	0.810	(0.736–0.883)								
MELD	B	0.815	(0.741–0.887)								
CLIF-C OFs	C	0.853	(0.794–0.913)								
CLIF-C ACLF	D	0.827	(0.761–0.895)								
MPM0-III	E	0.684	(0.602–0.765)								
SAP III	F	0.730	(0.653–0.808)								
APACHEII	G	0.744	(0.663–0.819)								
APACHEIII	H	0.841	(0.784–0.902)								

AUROC: area under receiver operating characteristic curve; The yellow color regions reflect significant difference in pairwise comparison at significance level α = 0.05 and is more important than the black ones.

**Table 4:** The optimal and futility cutoff values for APACHE III and CLIF-C ACLF scores to predict mortality and their sensitivity and specificity at different time points.

		APACH III	CLIF-C ACLF
**Optimal cutoff**		**79**	**47**
28th day	Sen	81.5%	87.0%
	Sp	60.4%	55.7%
90th day	Sen	76.2%	78.7%
	Sp	66.7%	59.3%
180th day	Sen	73.3%	75.5%
	Sp	70.8%	62.1%
365th day	Sen	70.0%	70.0%
	Sp	72.3%	64.2%
**Futility cutoff**		**125**	**71**
28th day	mortality	80.0%	80.0%
90th day	mortality	92.6%	80.0%
180th day	mortality	96.3%	90.0%
365th day	mortality	96.3%	90.0%

Sen: Sensitivity. Sp: Specificity. Optimal cutoff: assure over 70% sensitivity for four time points, and the specificity increases with time. Futility cutoffs: assuring that over 80% mortality would happen if the value exceeded the cutoff point for four time points.

## Supplementary Materials

The following are available online at https://www.mdpi.com/2077-0383/9/5/1540/s1, Table S1: The variables that each of the eight scores takes into consideration, Table S2: Demographics of 91 patients with ACLF and hepatitis B virus (HBV)-related liver cirrhosis admitted to the ICU.

## Abbreviations

INR	International normalized ratio
MAP	Mean arterial pressure
CPR	Cardiopulmonary resuscitation
SBP	Systolic blood pressure
HR	Heart rate
ICU	Intensive care unit
ACLF	Acute-on-chronic liver failure
ICU	Intensive care unit
LT	Liver transplantation
SOFAs	Sepsis organ failure assessment score
CLIF-SOFAs	Chronic liver failure SOFA score
CLIF-C OFs	CLIF consortium organ function score
CLIF-C ACLFs	CLIF consortium acute on chronic liver failure score
APACHE	Acute physiology and chronic health evaluation
MPM0-III	Mortality probability model III at zero hours
SAPs III	Simplified acute physiology score III
CTP	Child–Turcotte–Pugh score
MELDs	Model for end-stage liver disease score
EASL	European Association for the Study of the Liver
APASL	Asia-Pacific Association for the Study of the Liver
GCSs	Glasgow coma scale score
ROC	Receiver operating characteristic
AUROC	Area under the ROC curve
