# Supplementary material for: Comparing Eight Prognostic Scores in Predicting Mortality of Patients with Acute-On-Chronic Liver Failure Who Were Admitted to an ICU: A Single-Center Experience
Source: J Clin Med. 2020 May 20;9(5):1540. doi: 10.3390/jcm9051540 (PMC7290486; doi:10.3390/jcm9051540)
Supplement: Supplementary file 1 [file jcm-09-01540-s001.pdf]

**Supplementary Table 1.** The variables that each of the eight scores takes into consideration.

| Score | Variables |
|---|---|
| Child–Turcotte–Pugh (CTP) | Ascities, Encephalopathy, Bilirubin, Albumin, Prothrombin, Time |
| Model for End-stage Liver Disease (MELD) | Bilirubin, Creatinine, INR |
| CLIF Consortium Organ Function score (CLIF-C OFs) | Bilirubin, Creatinine, Encephalopathy, INR, MAP, Oxygenation (PaO2/FiO2) |
| CLIF Consortium Acute-on-Chronic Liver Failure score (CLIF-C ACLFs) | CLIF-C OFs, Age, WBC Count |
| Mortality Probability Model III at Zero Hours (MPM0-III) | Consciousness Level, Admission or Not, Prior CPR, Cancer, Chronic Renal Failure, Infection, Previous ICU Admission, Surgery before ICU Admission, SBP, HR, Age |
| Simplified Acute Physiology Score III (SAPs III) | Co-Morbidity †, Admission Days before ICU, Reason of ICU Admission ‡, Glasgow Coma Scales, Bilirubin, Body Temperature, Creatinine, Heart Rate, Arterial PH, Platelets, Systolic Blood Pressure, Oxygenation (PaO2/FiO2) |
| Acute Physiology and Chronic Health Evaluation II (APACHE-II) | Body Temperature, Mean Arterial Pressure, Heart Rate, Respiratory Rate, Oxygenation (FiO2), Arterial PH, Sodium, Potassium, Creatinine, Packed Cell Volume, WBC Count |
| Acute Physiology and Chronic Health Evaluation III (APACHE-III) | Pulse, Mean Blood Pressure, Body Temperature, Respiratory Rate, PaO2, AaDO2, Hematocrit, WBC Count, Creatinine, Urine Output, Blood Urine Nitrogen, Sodium, Albumin, Bilirubin, Glucose, Age, Comorbidities ††, Neurologic Abnormal (motor), Acid–Base disturbance |

† Cancer therapy, cancer, chronic heart failure (NYHA IV), cirrhosis, AIDS. ‡ Planned or unplanned ICU admission, surgical status at ICU, anatomical site of surgery, acute infection at ICU. †† AIDS, chronic heart failure (NYHA III, IV), lymphoma, metastatic cancer, leukemia/myeloma, immune compromised, cirrhosis.

**Supplementary Table 2.** Demographics of 91 patients with ACLF and hepatitis B virus (HBV)-related liver cirrhosis admitted to the ICU.

| Patients' Characteristics | All Patients (91 Patients) | Survivors (22 Patients) | Non-Survivors (69 Patients) | *p* Value |
|---|---|---|---|---|
| **Age (mean ± SD years)** | 55 ± 12 | 57 ± 14 | 59 ± 13 | <0.001 |
| **Gender = male** | 72 (79%) | 16 (73%) | 56 (81%) | 0.40 |
| **Clinical parameters:** | | | | |
| **Arterial PH** | 7.398 ± 0.126 | 7.440 ± 0.083 | 7.384 ± 0.185 | 0.069 |
| **PaO2/FiO2 200–300** | 73 (80%) | 19 (87%) | 54 (78%) | 0.318 |
| **PaO2/FiO2 < 200** | 18 (20%) | 3 (13%) | 15 (22%) | 0.005 |
| **MAP (mmHg)** | 89 ± 21 | 91 ± 14 | 89 ± 21 | 0.332 |
| **Temperature (℃)** | 36.6 ± 1.0 | 36.9 ± 1.0 | 36.4 ± 1.0 | 0.122 |
| **Respiratory rate (/min)** | 20 ± 5 | 19 ± 5 | 20 ± 5 | 0.212 |
| **Use of vasopressors** | 46 | 10 (45%) | 36 (59%) | 0.331 |
| **HE I-II** | 40 | 7 (32%) | 33 (49%) | 0.017 |

| | | | | |
|---|---|---|---|---|
| **HE III-IV** | 11 | 2 (9%) | 9 (13%) | 0.452 |
| **White cell count (×1000/μL)** | 11.0 ± 7.2 | 10.0 ± 6.2 | 11.3 ± 7.5 | 0.472 |
| **Hematocrit (mg/dL)** | 27.4 ± 6.5 | 29.9 ± 6.9 | 26.6 ± 6.1 | 0.035 |
| **INR** | 2.2 ± 1.0 | 1.7 ± 0.5 | 2.3 ± 1.0 | 0.015 |
| **Serum bilirubin (mg/dL)** | 9.8 ± 10.9 | 6.0 ± 7.9 | 11.0 ± 11.5 | 0.061 |
| **Serum creatinine (mg/dL)** | 2.0 ± 1.9 | 1.2 ± 1.0 | 2.2 ± 2.1 | 0.002 |
| **Serum sodium (mEq/L)** | 137 ± 7 | 138 ± 4 | 137 ± 8 | 0.031 |
| **Serum glucose (mg/dL)** | 193 ± 82 | 179 ± 85 | 198 ± 81 | 0.515 |
| **Albumin (g/dL)** | 2.6 ± 0.5 | 3.1 ± 0.5 | 2.5 ± 0.5 | <0.001 |
| **Mechanical ventilation use** | 29 | 5 (23%) | 24 (38%) | 0.236 |
